# Carpal coalition in school-aged children in Moray, Scotland: a retrospective radiographic case series

**DOI:** 10.1093/bjrcr/uaag044

**Published:** 2026-11-03

**Authors:** See-Yin Soo, Soniya Chauhan, Katrina Cruickshank, Mohamed Zubair Farook, Antonios Papadopoulos

**Affiliations:** Department of Orthopaedics, Dr Gray’s Hospital, Elgin IV30 1SN, United Kingdom; Department of Orthopaedics, Dr Gray’s Hospital, Elgin IV30 1SN, United Kingdom; Department of Orthopaedics, Dr Gray’s Hospital, Elgin IV30 1SN, United Kingdom; Department of Orthopaedics, Dr Gray’s Hospital, Elgin IV30 1SN, United Kingdom; Department of Orthopaedics, Dr Gray’s Hospital, Elgin IV30 1SN, United Kingdom

**Keywords:** carpal coalition, pediatric, wrist imaging

## Abstract

Carpal coalition is a congenital fusion between carpal bones, most often identified incidentally during radiographic evaluation following trauma. This retrospective case series describes the proportion and radiographic characteristics of carpal coalition in school-aged children within a defined geographic region of Moray, Scotland. A review of all Hand and Wrist radiographs of children aged 6-18 years old was performed over 2 years (2021 and 2022), including trauma/emergency and elective clinics. A total of 498 radiographs were analyzed, identifying 9 children with carpal coalition (proportion 1.8%). Hamate–capitate fusion was the most frequently observed pattern. All cases were incidental findings in children presenting with trauma, and none were clinically symptomatic. While this series provides descriptive insight into carpal coalition within a local Scottish cohort, the findings are not generalizable due to selection bias and the small sample size. Future research could help better understand why our observed proportion is higher.

## Introduction

Carpal coalition is a congenital anomaly characterized by the fusion of 2 or more carpal bones. It is present in approximately 0.1% of the population, based on historical studies (radiographic surveys).^1–3^ However, this condition is often under-recognized due to its predominantly asymptomatic presentation. Most cases are incidentally identified during radiographic evaluations following trauma.^1–3^ Carpal coalition can present as an isolated anomaly or in association with various syndromic or metabolic disorders.^3^ Isolated carpal coalitions typically involve bones within the same carpal row, whereas syndrome-related or post-infectious coalitions may involve bones in adjacent rows or the entire carpus. Idiopathic coalitions are more prevalent in females and African Americans, while the triquetrum-lunate coalition is the most commonly observed, occurring bilaterally in approximately 60% of the cases.^4^ This study aims to determine the proportion of carpal coalition in school-aged children with wrist radiographs within our community in Elgin, Scotland, and explore its potential clinical implications.

## Methods

A retrospective review of hand and wrist radiographs of school-aged children (6-18 years) within our catchment area was conducted over a 2-year period (January 2021-January 2023) using the Picture Archiving and Communication System (PACS) database. The PACS database was searched with filters applied to restrict the search to the Moray area, patients aged 6-18 years old, hand and wrist radiographs, and radiographs performed within the study period. Inclusion criteria included all hand and wrist radiographs performed in children aged 6-18 years during the study period, irrespective of clinical indication. Exclusion criteria included duplicate radiographs from the same encounter and incomplete or poor-quality radiographs preventing adequate assessment of the carpal bones.

Radiographs were reviewed for evidence of carpal coalition, defined as bony continuity between adjacent carpal bones or absence of a visible joint space. Standard posteroanterior and oblique trauma views were available; no specialized carpal views were performed. Findings were independently verified by 2 orthopedic consultants.

This study involved a retrospective review of anonymized radiographs. The NHS Research Ethics Committee was consulted on 22 November 2024 and confirmed that the project did not require formal ethical approval.

## Results

In 2022, our region included 46 primary schools with a total of 6824 primary school students and 5388 secondary school students, amounting to 12 212 subjects.^5^ A total of 252 hand and 464 wrist radiographs were reviewed. After filtering out 218 duplicates, 498 unique radiographs remained: 101 bilateral hand X-rays, 184 left-hand X-rays, and 211 right-hand X-rays. Nine patients (1.8%) demonstrated radiographic evidence of carpal coalition. The distribution was: hamate–capitate coalition (*n* = 6; see Figures 1 and 2), lunotriquetral coalition (*n* = 2), and pancarpal coalition (*n* =1; see Figure 3). Patient ages ranged from 9 to 18 years (mean 13.3 years), with a female predominance (2:1). All patients underwent radiographs following trauma (5 falls, 2 being hit by objects, and 1 fight). Among them, 2 had fractures not related to the carpus, and the remainder had soft tissue injuries related to the carpus. Thus, the coalition was an incidental radiographic finding. All patients were managed conservatively with analgesia, casting, or splinting, with no complications reported (Table 1).

**Figure 1:**
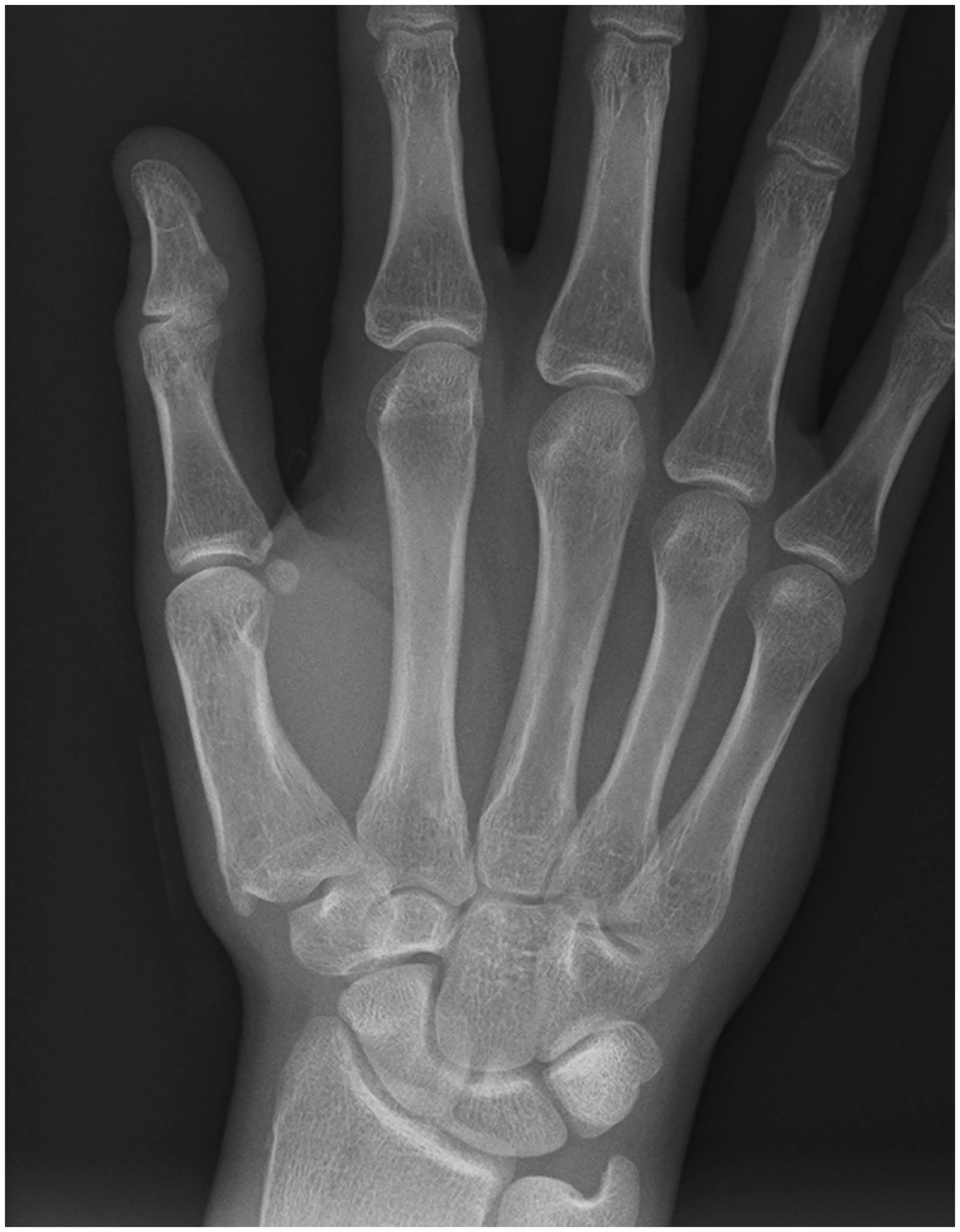
Posteroanterior (PA) radiograph of the hand demonstrating a mature hamate–capitate coalition (Case 5).

**Figure 2:**
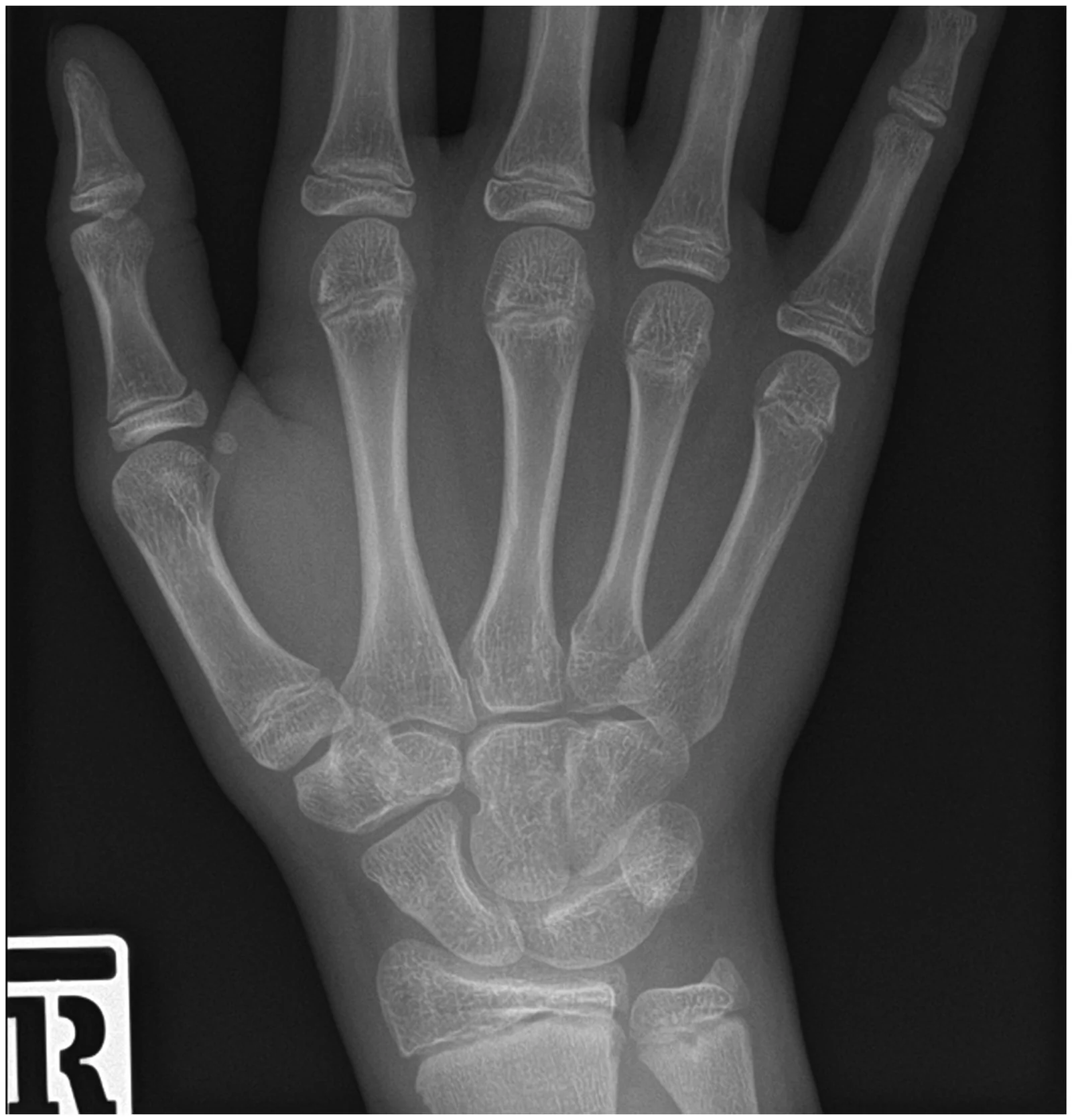
Posteroanterior (PA) hand radiograph in a skeletally immature patient highlighting a developing carpal coalition (Case 7).

**Figure 3:**
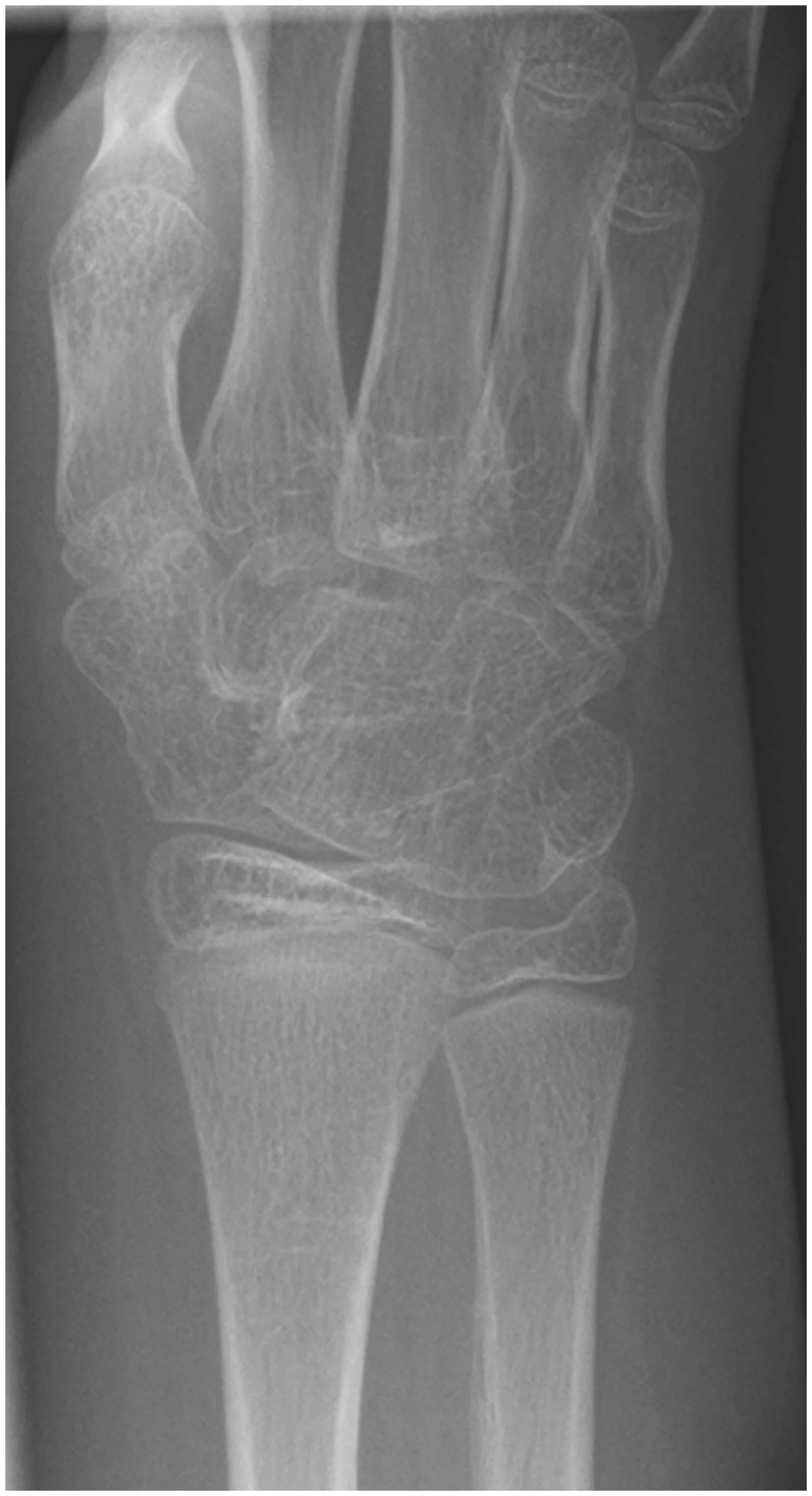
Radiographic view demonstrating a pancarpal coalition (Case 8).

**Table 1:** Summary of the 9 patients that demonstrated radiographic evidence of carpal coalition.

Case	Age	Sex	Laterality	Coalition type	Presentation	Injury	Management
**1**	11	M	Bilateral	Hamate–capitate	Fall onto glass	Soft tissue	Nil
**2**	16	F	Left	Lunotriquetral	Fight	Laceration to hand	Wound sutured
**3**	12	F	Left	Hamate–capitate	Fall playing football	Sprain/strain	Splint
**4**	11	F	Left	Lunotriquetral	Hit hand off desk	Sprain/strain	Splint
**5**	17	F	Right	Hamate–capitate	Fall onto hand	Fracture to second and third metacarpals	Cast
**6**	14	M	Right	Hamate–capitate	Fall onto hand	Distal radius fracture	Cast
**7**	14	M	Right	Hamate–capitate	Fall onto hand	Sprain/strain	Splint
**8**	11	F	Right	Pancarpal	Hit by football	Sprain/strain	Splint
**9**	14	F	Right	Hamate–capitate	Fall off horse	Sprain/strain	Splint

## Discussion

This retrospective case series identified carpal coalition in 1.8% of trauma-related pediatric radiographs, higher than classical anatomical estimates of 0.1% with variations up to 1.6% among different ethnic groups.^5^ However, this should not be interpreted as population prevalence due to significant selection bias. The true prevalence might not be possible to be calculated in modern days as it would be difficult to conduct a census study for a condition that is largely asymptomatic. The historical studies of 1971, which have been the baseline studies ever since, were thorough enough but may have been limited in their diagnostic ability due to the technology at the time to pick up subtle coalitions on radiographs. This has changed significantly with digital, high-resolution imaging nowadays. Subsequently, this present study may represent the closest one can get to a population prevalence. Artificial intelligence (AI) may shed some light in the future by picking up coalitions in asymptomatic patients and on a larger scale than this study. Inter-population anatomical studies report lunotriquetral coalition as the most common variant worldwide.^6^ The predominance of hamate–capitate coalition in this study is likely attributable to the small sample size rather than true regional variation. It is noteworthy that we observed a higher representation of females in our cohort, with twice as many females as males, aligning with existing reports that suggest idiopathic coalitions are more common in females.^6^

## Conclusion

Carpal coalition was incidentally identified in 1.8% of pediatric hand and wrist radiographs in a Scottish district hospital. To better understand why our observed proportion is higher, future research could include an expanded geographic scope: conducting multicenter studies across different regions to identify potential geographic or environmental factors influencing prevalence. The use of AI might shed some more light on this.

These approaches could help calculate the exact prevalence of this condition and possibly clarify the factors contributing to the higher proportion observed in our study, enhancing the understanding of carpal coalition’s epidemiology and clinical significance.
